# Simply Versatile: The Use of *Peribacillus simplex* in Sustainable Agriculture

**DOI:** 10.3390/microorganisms11102540

**Published:** 2023-10-12

**Authors:** Julia Manetsberger, Natacha Caballero Gómez, Carlos Soria-Rodríguez, Nabil Benomar, Hikmate Abriouel

**Affiliations:** 1 Area of Microbiology, Department of Health Sciences, Faculty of Experimental Sciences, University of Jaén, 23071 Jaén, Spain; 2 Area of Public International Law and International Relations, Department of Public and European Common Law, University of Jaén, 23071 Jaén, Spain

**Keywords:** *Peribacillus simplex*, antimicrobial activity, sustainable agriculture, bioremediation, European Green Deal

## Abstract

*Peribacillus simplex* is a Gram-positive, spore-forming bacterium derived from a vast range of different origins. Notably, it is part of the plant-growth-promoting rhizobacterial community of many crops. Although members of the *Bacillaceae* family have been widely used in agriculture, *P. simplex* has, so far, remained in the shadow of its more famous relatives, e.g., *Bacillus subtilis* or *Bacillus thuringiensis*. Recent studies have, however, started to uncover the bacterium’s highly promising and versatile properties, in particular in agricultural and environmental applications. Hence, here, we review the plant-growth-promoting features of *P. simplex*, as well as its biocontrol activity against a variety of detrimental plant pests in different crops. We further highlight the bacterium’s potential as a bioremediation agent for environmental contaminants, such as metals, pesticide residues, or (crude) oil. Finally, we examine the recent developments in the European regulatory landscape to facilitate the use of microorganisms in plant protection products. Undoubtedly, further studies on *P. simplex* will reveal additional benefits for agricultural and environmentally friendly applications.

## 1. Introduction

Sustainable agriculture is key in ensuring a continuous food supply for the growing world population, while at the same time minimizing negative effects on the environment [1]. This is also reflected in policy developments such as the European Green Deal and its ambitious Farm to Fork strategy, aiming at halving the use and risk of chemical pesticides and increasing organic farming practices [2]. 

One promising approach to replacing chemical products is the use of soil microbial inoculants, which are predominantly based on plant-growth-promoting (PGP) fungi and bacteria [3,4]. When applied to soil and/or plants, these microorganisms can exert several beneficial effects on their surroundings, such as (i) acting as biopesticides, (ii) enhancing plant growth, or (iii) improving soil conditions (e.g., through bioremediation or bioadsorption) [3]. Thus, bacterial inoculants can increase agronomic efficiency by reducing production costs and environmental pollution, as well as (partially) eliminating the use of chemical fertilizers and pesticides [5]. 

Plant-growth-promoting bacteria (PGPB) and plant-growth-promoting rhizobacteria (PGPR) are essential parts of the soil microbiome, sustaining plant health and growth. These microorganisms colonize the soil, plant rhizosphere, and root surface or interior and fulfil a variety of useful functions, such as increasing nutrient availability, counteracting abiotic stress, or improving the stress tolerance of the plant [5,6,7,8]. Here, members of the genus *Bacillus*—and recently reclassified closely related genera [9]—are one of the predominant microbial communities and play an important role in maintaining healthy soils conducive for plant growth and nutrition [8,10]. These Gram-positive bacteria are characterized by their ability to form dormant endospores, enabling them to withstand harsh conditions otherwise fatal to vegetative cells [11,12,13]. In addition, their ability to produce a wide arsenal of biologically active compounds with inhibitory and/or plant-growth-promoting effects has been well documented [14,15,16].

The biocontrol activity of a microorganism can generally be classified into two mechanisms. Direct antimicrobial activity includes the synthesis of phytohormones, as well as the production of antibiotics, hydrolytic enzymes, or lipopeptides [17]. In this regard, *Bacillus* spp. have been recognized as promising sustainable plant protection agents presenting a viable alternative to chemical pesticides [18], with, e.g., *B. thuringiensis*-, *B. subtilis*-, and *B. amyloliquefaciens*-containing formulations already commercially available [17,19,20]. 

Indirect mechanisms of biocontrol activity include (amongst others) inducing systemic resistance (ISR) in plants [17], which activates/increases plants’ resistance towards phytopathogenic infections and indirectly stimulates plant growth [21,22]. Here, *Bacillus* spp. can induce systemic resistance through different mechanisms, such as the secretion of enzymes, cyclic lipopeptides, or volatile organic compounds (VOC) [23]. That said, it is important to note that there is no clear separation of ISR and antimicrobial activity, as several antimicrobial lipopeptides, e.g., fengycin and surfactin, or VOCs can simultaneously induce systemic resistance [24]. 

Although positive environmental impacts of members of *Bacillus* spp. and related genera have been widely demonstrated, studies on *Peribacillus simplex* have only recently started to uncover the bacterium’s wide range of highly promising PGP features, including the ability to promote plant growth through nutrient fixation, the production of antimicrobial compounds, or acting as biosorbent for environmental contaminants. Hence, here, we provide a comprehensive overview of these findings and highlight *P. simplex*’s potential for its use in sustainable agricultural. Finally, with a view towards the future applications of *P. simplex* as a biocontrol agent, we will briefly summarize the requirements and changes in the European Regulation in the light of the European Green Deal and Farm to Fork strategy, which aim to facilitate the use of microorganisms in plant protection products. 

## 2. Genus Peribacillus

Members of the genus *Peribacillus* belong to the family of *Bacillaceae* and are rod-shaped, Gram-positive, endospore-forming bacteria. Aerobic or facultative anaerobic bacteria were previous members of the genus *Bacillus*, however, after an extensive taxonomic reclassification in 2020 using phylogenomics and comparative genomic analyses, the species have been rearranged based on molecular markers to form a separate monophylogenetic group of the genus *Peribacillus* [9,25]. Today, the genus includes 21 species, with *Peribacillus simplex* as the type strain (Table 1) [26]. 

Many of the species have been originally isolated from soil and plant samples, although they can be derived from a wide variety of origins, such as near the Viking spacecraft at Kennedy Space Center [27] or stratospheric air samples at a 41 km altitude [28].

**Table 1:** Members of the *Peribacillus* genus. Original sources of isolation are indicated.

*Peribacillus* Species [25,26]	Original Isolation Source	Ref.
*Peribacillus acanthi*	Rhizosphere soil of a mangrove plant *Acanthus ilicifolius*	[29]
*Peribacillus alkalitolerans*	Marine sediment near a hydrothermal vent	[30]
*Peribacillus asahii*	Soil	[31]
*Peribacillus butanolivorans*	Soil	[32]
*Peribacillus castrilensis*	River otter	[33]
*Peribacillus cavernae*	Cave soil	[34]
*Peribacillus deserti*	Desert soil	[35]
*Peribacillus endoradicis*	Soybean root	[36]
*Peribacillus faecalis*	Cow feces	[37]
*Peribacillus frigoritolerans*	Arid soil	[38,39]
*Peribacillus glennii*	Vehicle assembly building at Kennedy Space Center	[27]
*Peribacillus gossypii*	Stem of *Gossypium hirsutum*	[40]
*Peribacillus huizhouensis*	Paddy field soil	[41]
*Peribacillus kribbensis*	Soil	[42]
*Peribacillus loiseleuriae*	Soil from a loiseleuria plant	[43]
*“Peribacillus massiliglaciei”* ^1^	Siberian permafrost	[44]
*Peribacillus muralis*	Deteriorated mural paintings	[45]
*Peribacillus psychrosaccharolyticus*	Soil or lowland marsh.	[46]
*Peribacillus saganii*	Vehicle assembly building at Kennedy Space Center	[27]
*Peribacillus simplex*	Soil	[46]
*Peribacillus tepidiphilus*	Tepid spring	[47]

^1^ Nomenclature status not validly published.

## 3. Plant-Growth-Promoting Properties 

Members of the *Bacillus* genus (as traditionally defined) are among the most widespread Gram-positive soil microorganisms and are predominant in the plant-growth-promoting bacteria (PGPB) community [10]. The beneficial effects of the family members have been well documented [8,10,18]. 

In this regard, a number of studies have highlighted *P. simplex*’s potential to act as a plant-growth-promoting microorganism (Table 2).

### 3.1. Plant Growth Promotion through Compound Secretion

With the aim of searching for sustainable plant supplements or alternatives to chemical fertilizers, the use of PGPB has shown great potential, minimizing environmental impacts [51]. *P. simplex* demonstrates a broad range of activity, stimulating growth in a large variety of commercially relevant crops, such as tomato, wheat, soybean, or corn (Table 2), and has sometimes achieved over a quarter of crop yield increase [57]. In some cases, growth stimulation can notably reach levels similar to chemical fertilizers, making the bacterium a sustainable alternative to potentially harmful chemicals in food production [50]. Growth stimulation has most commonly been attributed to direct growth promotion via auxin production (indole-3- acetic acid, IAA) or siderophore secretion [22,50,56,58].

Another way of stimulating plant growth is the emission of volatile organic compounds (VOC), e.g., acetoin and 2,3-butanediol. When emitted by PGPB bacteria, these compounds can act as plant growth promotion triggers [52]. Gutiérrez-Luna et al. suggested that the VOCs secreted by *P. simplex* isolated from lemon plants improved the root growth and development in *Arabidopsis thaliana* under greenhouse conditions [52]. These compounds, mostly ketones and aldehydes also with antimicrobial attributes, included 2-nonenal, benzaldehyde, acetophenone, 6,10,14-trimethyl-2-pentadecanone, and 1-butanol, amongst others. However, there was no direct, experimental support for the effect of specific VOCs on plant growth promotion [52]. 

Finally, recent studies have shown that these growth promotion effects can be maximized when using combined inoculations with other PGPBs [51] or inorganic material [58]. This effect was particularly visible when combining PGP bacteria (*P. simplex*) and nitrogen (N)-fixating rhizobacteria (*B. subtilis*, *Rhizobium leguminosarum* bv. *Viciae*) in peas [53], while *P. simplex*-based bioformulations showed hydrogen cyanide (HCN), siderophore, and ammonia production in wheat [49]

In contrast, studies investigating the addition of inorganic acids such as salicylic acid together with *P. simplex* did not show any effect on plant growth [61].

### 3.2. Improved Nutrient Availability

Recent research attempts have aimed at increasing the concentrations of specific nutrients or micronutrients, thus improving plant health and nutritional value [4]. Although many techniques are based on plant-breeding techniques or transgenics, the use of PGP bacteria could also boost the uptake of specific nutrients in crops. 

Studies have shown that siderophore-producing *P. simplex* can increase the uptake of iron in potatoes, while at the same time improving overall plant growth and yield [4]. 

*P. simplex* isolates have also demonstrated a high phosphate and zinc solubilization index in wheat [49], whereas high phosphate solubilization was detected in experiments with tomato plants. The latter, however, was distinctly strain-dependent [50]. 

Given that, in the soil, microorganisms occur in communities presumably acting synergistically, the combination of several PGPBs has shown better plant growth promotion effects than when used in isolation [3]. For example, co-culturing canola plants with *P. simplex* improved the shoot and root weight, in addition to enhancing the molybdenum micronutrient uptake [48]. Higher soluble nutrient concentrations (phosphate, magnesium, manganese, and sulfur), as well as increased phosphate uptake, could be obtained in winter wheat upon co-inoculation of the soil fungus *Penicillium bilaiae* with *P. simplex* (isolated from *P. biliaiae*) [55]. Equally, co-culturing *P. simplex* with inorganic silicon (Si) could improve the phosphate (P) uptake from P-rich and P-deficient soils. This was attributed to reduced oxidative stress as a result of increased antioxidant enzyme production, ultimately lowering the environmental stress for the plant and preventing root deterioration.

### 3.3. Root Colonization

PGPRs colonize the soil closely surrounding plant roots (rhizosphere), where they exert beneficial effects on plants. Hence, the success of microorganisms used as inoculants in agricultural crops greatly depends on the ability to colonize the host plant roots and body and prevail against other competing microorganisms [5,62]. The successful association of the bacteria with the plant roots is achieved by chemotaxis, attachment, and distribution along the roots. Once established, the bacterial colony size will determine and improve the root coverage and antagonism [62].

*P. simplex* has demonstrated a good root colonization potential and persistence in several commercial plants, such as wheat, tomato, and pine tree roots [51,55,62]. In some cases, *P. simplex* showed a higher rate of colonization than other *Bacillus* species (e.g., *B. subtilis*) [49]. Fluorescent localization studies with the transgenic *P. simplex* strain S11R41 isolated from pine tree rhizosphere have, in particular, confirmed that the bacterium is able to rapidly associate with tree roots, forming clusters at emerging lateral roots and elongation zones [62]. 

Regarding biofilm formation, GFP-report localization studies have not evidenced any biofilm formation of *P. simplex* associated with tree roots [62]. 

## 4. Biocontrol Activity

*P. simplex* strains isolated from different environments showed biocontrol activity against a large range of phytopathogens, mostly fungi, but also nematodes and bacteria, which was detected in several commercially highly relevant plants, such as potato, wheat, or tobacco (Table 3). 

### 4.1. Antimicrobial Activity

The antifungal activity of *P. simplex* has been demonstrated in a number of studies, most of them conducted on the phytopathogenic fungus *Fusarium* spp. In vitro assays showed up to a 70% growth inhibition of the plant pest and fungal hyphal thinning [17,53,63], however, compared to *B. subtilis*, the effects were slightly lower [17]. In planta experiments further confirmed these antifungal properties, greatly reducing disease severity after *P. simplex* application to the root seedlings of row crops or in black cumin [54,63]. The authors cautioned, however, that the results obtained from in vivo and in vitro antagonistic assays were not always aligned [63], and thus appropriate care should be taken for the screening of biocontrol agents under field conditions. Schwartz et al. [53] also confirmed *P. simplex*’s antagonistic activity against *Fusarium* spp., which was, however, dependent on growth conditions. This study was of particular interest, as it demonstrated the combined antimicrobial and plant-growth-promoting effects of *P. simplex* isolate 30N-5 in pea (Table 2 and Table 3), suggesting that such a combined activity could be more effective under field conditions [53]. Similar results were observed for isolates PHYB1 and PHYB9 in black cumin treatment [54]. Regarding the mode of action, in silico genomic studies indicated the presence of genes involved in the chitin degradation pathway and hydrolytic enzyme production, as well as cell-wall-degrading enzymes such as cellulase, pectinase, and xylanase, all of which are indicators for *P. simplex*’s antimicrobial activity [17]. Scanning electron microscopy studying the interaction between *P. simplex* and *F. camptoceras* demonstrated the bacterial adhesion to the fungus and the colonization of hyphae, causing tissue maceration [54].

*P. simplex* also reduced fungi-associated diseases in potato (pink rot) and wheat (Septoria Tritici Blotch) [65,66], while other studies demonstrated its antagonistic activity against the phytopathogens *Pectobacterium* sp. and *Xylella fastidiosa* [67,68]. Finally, in silico studies of the strain BA2H3 suggested the production of the antimicrobial compounds bacitracin and anthrachelin [67,74].

Regarding VOCs, several studies have highlighted *P. simplex*’s ability to produce a variety of microbial volatile organic compounds, including 2-ethyl-3,5-dimethylpyrazine, phenol, 1-decanol, 2-propanone, and benzaldehyde [17,64]. In this regard, Gu et al. [64] showed that soil-derived *P. simplex* strains secreted a mix of volatile organic compounds from the phenol, alcohol, aldehyde ketone alkyl, alkene, acid, ether, or heterocyclic groups, with strong antagonistic activity against the parasitic nematodes *Panagrellus redivivus* and *Bursaphelenchus xylophilus*. One important consideration with regard to the use of bacterial VOCs is that this mix is potentially less likely to select for resistance upon fumigation treatment.

### 4.2. Systemic Resistance

Recent studies have indicated that, besides antifungal activity in tobacco plants, pre-treatment with the *P. simplex* strain HS-2 increased reactive oxygen species (ROS) production and lowered plant cell wall permeability through increased callose production in response to a pathogen challenge [23]. Both reactions are indicators of the plant immune response. In addition, priming with this strain enhanced the expression of plant-related defense genes (e.g., lipoxygenase), as well as MAPK (mitogen-activated protein kinases) signals [23]. 

Fungal antagonism was also demonstrated in vitro and in planta against the forest fungal pathogens *Heterobasidion annosum* s.s., *Armillaria mellea*, and *Fusarium circinatum*. Notably, the treatment of pine seedlings with *P. simplex* considerably reduced lesions and plant mortality after pathogen exposure, which was tentatively attributed to antibiosis/systemic response [69,70]. Here, a dual application of the bacterium together with essential oils able to reduce seedling lesions was suggested as a plant prophylactic treatment [70]. 

Several studies by Yu-xi Duan and colleagues furthermore demonstrated the antagonistic effects of *P. simplex* Sne545 against nematodes through the activation of induced systemic resistance in soybean using a wide range of different analytical approaches [71,72,73]. First, metabolomic and transcriptomic analyses showed that the bacterium induces ISR by modulating the accumulation of nematocidal compounds (4-vinylphenol, L-methionine, piperine, and palmitic acid) after root infection, hence improving soybean resistance against pathogenic attacks [72]. Then, additional ISR-active compounds were determined using ^1^H-NMR and ^13^C-NMR as cyclic (Pro-Tyr), phenylalanine, cyclic (Leu-Pro), uracil, cyclic (Val-Pro), and tryptophan. The latter three notably activated the root resistance pathways (SA and JA pathways) in the plant [71]. Finally, metabolomics studies identified 15 metabolites involved in nematode resistance as a result of *P. simplex* Sne545 priming. These metabolites were involved in the provision of nematode nutrient sources (glucose, fructose, sucrose, and trehalose), the production of nematocidal compounds (melibiose and gluconic acid, lactic acid, phytosphingosine, and noradrenaline), and improved disease resistance (oxoproline, maltose, and galactose) [73]. 

Studies on wild rice have furthermore highlighted that pretreatment with strain 499G2 can promote plant growth (mostly through IAA production), while at the same time inducing plant resistance [60]. Overall, this is a good illustration that systemic resistance in plants (and bacterial antagonistic activity) mostly consists of an elaborate interplay of different pathways and compounds warding off the phytopathogen and often simultaneously improving plant resistance, survival, and health [60,71,72,73]. 

## 5. Biosorption and Bioremediation

**Figure 1:**
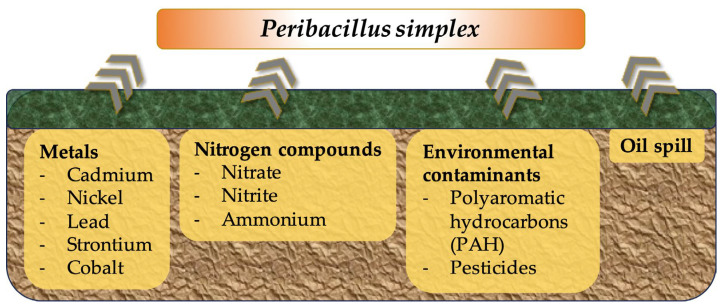
Schematic representation of bioadsorption and bioremediation activity of *P. simplex*.

The use of microorganisms as a remedy for contaminated zones is widely accepted. Several microorganisms have shown good potential as biosorbents for binding metals, environmental contaminants, or even mineral oil, immobilizing the contaminating substances and hindering their entry into the plant, food chain, or ground water [75,76,77]. In addition, bioremediation by microorganisms can indirectly promote plant growth by reducing stress conditions. In this regard, studies throughout the years have shown the bioremediation activity of *P. simplex* (Figure 1). 

Early studies performed in the 1990s revealed that *P. simplex* could remove metals from contaminated soils and thus act as an environmental decontamination agent [75]. The bacterial uptake of cationic metal is usually attributed to interactions with the negatively charged cell wall. In particular, *P. simplex*’s ability to adsorb heavy metals showed a pH dependency with an optimum performance close to a neutral to alkaline pH. Researchers thus concluded that metal uptake was dependent on variably charged protonation sites (e.g., amino groups, phosphate, or carboxylate) [75]. As an example, Valentine and colleagues showed how the *P. simplex* strain ZAN-44 can adsorb divalent cadmium, nickel, cobalt, and strontium ions with a higher efficiency than *B. subtilis* 168 or *Escherichia coli* K-12. Notably, the latter two of the tested ions (^60^Co and ^90^Sr) were radionuclides, making *P. simplex* an interesting biosorbent for the cleaning of radioactively contaminated sites [75]. The ability of *P. simplex* to adsorb lead has been demonstrated in the literature, while authors have suggested that the bacterium could be exploited for bioremediation purposes [76]. Elevated levels of cadmium have been a major concern also in cocoa plants, with many initiatives aiming at reducing cadmium levels. Here, *P. simplex* has been proven as a highly promising sustainable biosorbent material for removing cadmium from contaminated soils and preventing its entry into plants and food chains [78].

Bioremediation activity has also been shown for other environmental contaminants such as low-molecular-weight polyaromatic hydrocarbon fluorene and phenanthrene, as well as nitrate, nitrite, and ammonium [79,80,81]. In particular, nitrogen removal capacity was favored by the strains’ (*P. simplex* H-b) tolerance of low temperatures [81].

The pesticidal burden of soils has become an increasing concern in agriculture and the food industry, given the long-time stability and non-specific toxicity of many active substances [82]. In this regard, several studies have demonstrated *P. simplex*’s ability to remove chemical pesticides from contaminated soils, as shown with the example of chlorsulfuron [83].

Finally, *P. simplex* isolates derived from bioaugmented oil contaminated soil have been classified as hydrocarbonoclastic bacteria, i.e., able to live on hydrocarbons as an energy source [84]. In addition to biodegradation, a *P. simplex* strain isolated from oil-contaminated sea sediment showed a high oil recovery efficiency through the production of a lipopeptide surfactant, including at a high salinity [85]. These features make *P. simplex* a particularly interesting candidate for the bioremediation of (crude) oil-contaminated sites via oil degradation and recovery. 

## 6. EU Regulatory Aspects on the Use of Microorganisms in Sustainable Agriculture

Plant pathogens present a serious threat to agricultural productivity and can cause severe crop loss. For decades, chemical pesticides have been used to fight phytopathogens, including bacteria, fungi, or insects. However, with regulatory and food safety requirements becoming much stricter, a switch towards sustainable agriculture using biological alternatives to hazardous chemicals is gaining importance. In this regard, *P. simplex* and other members of the *Bacillaceae* family have shown promising traits that could be exploited in commercial agriculture, thus providing solutions to recent policy requests. Here, with the aim of contributing towards the objectives set under the Farm to Fork Strategy to reduce the overall use and risk of chemical pesticides by 50% and the use of more hazardous pesticides by 50% by 2030 [2], the European Union (EU) is facilitating the application of microorganisms in plant protection products. More specifically, it has developed four implementing regulations—applicable since 2022—regarding the approval of microorganisms as active substances in plant protection products (PPP). The first modification was Commission Regulation (EU) 2022/1438 amending Annex II of Regulation (EC) No 1107/2009 [86,87]. The latter provides rules for the authorization of PPPs and their placing on the market, while the amendment (amongst others) extends specific criteria related to microorganisms. Some of these main modifications and/or additions specifically refer to the requirement that the microorganism in question needs to be deposited at an internationally recognized culture collection and receive an accession number. It must be identified at minimum at the strain level and information must be provided about whether the biological materials are wild types, mutants, or genetically modified organisms. Regarding the safety aspects of the microorganisms, they must not be pathogenic to humans and must have no known functional and transferable gene coding for resistance to relevant antimicrobial agents. In this regard, the amendment further requires the microorganism to be susceptible to at least two classes of antimicrobial agents for it to be considered a low-risk active substance [87].

Other amendments related to the necessary information to be submitted for active substances and the specific data requirements for microorganisms were Commission Regulation (EU) 2022/1439 amending Regulation (EU) No 283/2013 [88,89]. We particularly highlight a modification referring to antimicrobial resistance (AMR), as well as the presence of antimicrobial resistance genes (ARG) [88]. Here, information is required on whether the bacterium shows any resistance to relevant antimicrobial agents or if ARG are acquired, transferable, and functional. These changes also relate to modifications in the data requirements for plant protection products containing microorganisms, as reflected in Commission Regulation (EU) 2022/1440 amending Part B of the Annex to Regulation (EU) No 284/2013 [90,91]. Thus, both amendments aim to update the data requirements for the latest scientific developments and adapt them to the specific biological properties of microorganisms. 

Finally, given the abovementioned updated regulatory documents, Commission Regulation (EU) 2022/1441 amends Regulation (EU) No 546/2011 regarding the uniform principles for the evaluation and authorization of plant protection products containing microorganisms. Hence, data assessments are aligned across Member States, ensuring a high level of protection for human and animal health [92,93]. 

## 7. Conclusions

The advantages of *Bacillus* spp. in agriculture have long been recognized. That said, *Peribacillus simplex* has not received as much attention as other strains in this regard. However, recent efforts focusing on this spore former have shown its various beneficial effects for agricultural and environmental applications. These notably include plant-growth-promoting properties and excellent root colonization skills, as well as antimicrobial compound production and the induction of the plant systemic immune response. Regarding environmental functions, studies have begun to reveal highly promising properties of *P. simplex* as a bioremediation agent, for example, of heavy metals, pesticides, or oil removal and recovery. Future work will surely uncover further modes of action for this versatile bacterium. 

A revision of the European regulatory landscape highlights changes in the legal frameworks to facilitate the use of microorganisms in sustainable plant protection products, while imposing strict safety rules to protect humans, animals, and the environment. 

## Figures and Tables

**Table 2:** Examples of uses of *Peribacillus simplex* as plant-growth-promoting bacteria.

*P. simplex* Isolate	Effect	Tested Plant	Ref.
MRBN26	Increased shoot and root weight	Canola plant	[48]
KY604953	Enhanced germination, root growth, and nutrient uptake	Wheat	[49]
K10	Improved plant height, tuber weight, photosynthesis yield, transpiration rate, water use efficiency, and overall yield	Potato	[4]
MH671854.1, MH671861.1	Increased shoot and root weight, IAA production, and high phosphate solubilization	Tomato	[50]
KBS1F-3	Increased shoot and root weight, IAA production, and high phosphate solubilization	Tomato and wheat	[51]
KY515398	Stimulation of root and shoot growth	Corn, wheat, and soybean	[22]
L266	Stimulation of primary root growth and lateral root development	*Arabidopsis thaliana*	[52]
30N-5	Increases number of lateral roots	Pea legume	[53]
PHYB1; PHYB9	Increased root and foliar dry weight	Black cumin	[54]
313, 371	Increased phosphate uptake and increased soil nutrient concentrations (co-cultured with *P. biliaiae*)	Winter wheat	[55]
RC19	Root induction	Kiwi	[56]
SYM00260	Increased yield and root and shoot dry weight	Corn and soybean	[57]
UT1	Improved phosphate, potassium, and silica uptake, and increased root and shoot biomass	Wheat	[58]
EGE-B-1.2.k	High phosphate solubilization	Tomato, pepper, and eggplant	[59]
499G2	Increased nitrogen, phosphorus, And IAA in plant leaves	Wild rice	[60]

**Table 3:** Applications of *Peribacillus simplex* as biocontrol agent in selected crops/diseases and associated phytopathogens. Studies on the species’ antimicrobial activity, as well as the induction of the plant systemic response, are considered.

*P. simplex* Isolate	Effect	Target	Class	Test Conditions (Plant) *	Ref.
**Antimicrobial activity**					
30N-5; 11; 237	Presence of biocontrol genes/cellulase, xylanase, pectinase, and chitinase production	*Fusarium* spp.	Fungus	In vitro/In silico	[17]
30N-5	Pathogenetic growth inhibition			In vitro	[53]
R180	Pathogenetic growth inhibition and reduction in disease severity			In vitro and in planta (corn, wheat, and soybean)	[63]
PHYB1 and PHYB9	Reduction in disease severity, and hyphal tissue maceration			In vitro and in planta (black cumin)	[54]
Isolate 1–6	VOC production	*Panagrellus redivivus* and *Bursaphelenchus xylophilus*	Nematode	In vitro	[64]
Alg.24B2	Production of lytic enzymes and lipopetides	*Zymoseptoria tritici*	Fungus	In vitro	[65]
03WN13; 03WN23;03WN25	Reduced lesion size and disease (pink rot)	*Phytophthora erythroseptica*	Fungus	In planta (potato)	[66]
BA2H3	Pathogenetic growth inhibition and reduction in soft rot symptoms	*Pectobacterium* sp.	Bacterium	In vitro and in planta (potato)	[67]
UJA_MA_369	Pathogenetic growth inhibition	*Xylella fastidiosa*	Bacterium	In vitro	[68]
**Induced Systemic Resistance**					
HS-2	Antifungal/ increased ROS and callose production	*Pythium aphanidermatum*	Phytium	In vitro and in planta (tobacco)	[23]
499G2	Increased antioxidant enzyme production	*Magnaporthe grisea*	Fungus	In vitro and in planta (wild rice)	[60]
S11R41	Reduced lesions and plant mortality	*Heterobasidion annosum* and *Armillaria* *mellea*	Fungus	In vitro and in planta (*Pinus radiata*)	[69]
	Reduced fungus growth and density and reduced lesion length	*Fusarium circinatum*			[70]
Sneb545	Increased plant resistance, reduced infection/nematode penetration, and reduced nematode growth	*Heterodera glycines*	*Nematode*	In vitro and in planta (soybean seeds)	[71,72,73]

* Test conditions indicate if studies were performed in vitro, in silico, or the tested plant in case of in planta tests.

## Data Availability

Not applicable.

## References

[1] Velten S., Leventon J., Jager N., Newig J. (2015). What Is Sustainable Agriculture? A Systematic Review. Sustainability.

[2] European Commission (2020). Communication from the Commission to the European, the Council, the European Economic and Social and the Committee of the Regions—A Farm to Fork Strategy for a Fair, Healthy and Environmentally-Friendly Food System.

[3] O’Callaghan M., Ballard R.A., Wright D. (2022). Soil Microbial Inoculants for Sustainable Agriculture: Limitations and Opportunities. Soil Use Manag..

[4] Mushtaq Z., Nazir A., Asghar H.N., Zahir Z.A. (2022). Interactive Effect of Siderophore-Producing Bacteria and l-Tryptophan on Physiology, Tuber Characteristics, Yield, and Iron Concentration of Potato. Potato Res..

[5] de Souza R., Ambrosini A., Passaglia L.M.P. (2015). Plant Growth-Promoting Bacteria as Inoculants in Agricultural Soils. Genet. Mol. Biol..

[6] Saxena A.K., Kumar M., Chakdar H., Anuroopa N., Bagyaraj D.J. (2020). Bacillus Species in Soil as a Natural Resource for Plant Health and Nutrition. J. Appl. Microbiol..

[7] Glick B.R. (1995). The Enhancement of Plant Growth by Free-Living Bacteria. Can. J. Microbiol..

[8] Manetsberger J., Caballero Gómez N., Benomar N., Christie G., Abriouel H. (2023). Characterization of the Culturable Sporobiota of Spanish Olive Groves and Its Tolerance toward Environmental Challenges. Microbiol. Spectr..

[9] Gupta R.S., Patel S., Saini N., Chen S. (2020). Robust Demarcation of 17 Distinct Bacillus Species Clades, Proposed as Novel Bacillaceae Genera, by Phylogenomics and Comparative Genomic Analyses: Description of *Robertmurraya kyonggiensis* Sp. Nov. and Proposal for an Emended Genus Bacillus Limiting It Only to the Members of the Subtilis and Cereus Clades of Species. Int. J. Syst. Evol. Microbiol..

[10] Radhakrishnan R., Hashem A., Abd Allah E.F. (2017). Bacillus: A Biological Tool for Crop Improvement through Bio-Molecular Changes in Adverse Environments. Front. Physiol..

[11] Setlow P. (2006). Spores of Bacillus Subtilis: Their Resistance to and Killing by Radiation, Heat and Chemicals. J. Appl. Microbiol..

[12] Nicholson W.L. (2002). Roles of Bacillus Endospores in the Environment. Cell. Mol. Life Sci..

[13] Driks A. (1999). Bacillus Subtilis Spore Coat. Microbiol. Mol. Biol. Rev..

[14] Abriouel H., Franz C.M.A.P., Ben Omar N., Galvez A. (2011). Diversity and Applications of Bacillus Bacteriocins. FEMS Microbiol. Rev..

[15] Caulier S., Nannan C., Gillis A., Licciardi F., Bragard C., Mahillon J. (2019). Overview of the Antimicrobial Compounds Produced by Members of the Bacillus Subtilis Group. Front. Microbiol..

[16] Vlajkov V., Pajčin I., Loc M., Budakov D., Dodić J., Grahovac M., Grahovac J. (2022). The Effect of Cultivation Conditions on Antifungal and Maize Seed Germination Activity of Bacillus-Based Biocontrol Agent. Bioengineering.

[17] Khan N., Martínez-Hidalgo P., Ice T.A., Maymon M., Humm E.A., Nejat N., Sanders E.R., Kaplan D., Hirsch A.M. (2018). Antifungal Activity of Bacillus Species against Fusarium and Analysis of the Potential Mechanisms Used in Biocontrol. Front. Microbiol..

[18] Miljaković D., Marinković J., Balešević-Tubić S. (2020). The Significance of Bacillus Spp. in Disease Suppression and Growth Promotion of Field and Vegetable Crops. Microorganisms.

[19] Kumar P., Kamle M., Borah R., Mahato D.K., Sharma B. (2021). *Bacillus thuringiensis* as Microbial Biopesticide: Uses and Application for Sustainable Agriculture. Egypt. J. Biol. Pest Control.

[20] Villarreal-Delgado M.F., Villa-Rodríguez E.D., Cira-Chávez L.A., Estrada-Alvarado M.I., Parra-Cota F.I., De los Santos-Villalobos S. (2018). The Genus Bacillus as a Biological Control Agent and Its Implications in the Agricultural Biosecurity. Rev. Mex. De Fitopatol. Mex. J. Phytopathol..

[21] Santoyo G., del Orozco-Mosqueda M.C., Govindappa M. (2012). Mechanisms of Biocontrol and Plant Growth-Promoting Activity in Soil Bacterial Species of Bacillus and Pseudomonas: A Review. Biocontrol. Sci. Technol..

[22] Akinrinlola R.J., Yuen G.Y., Drijber R.A., Adesemoye A.O. (2018). Evaluation of Bacillus Strains for Plant Growth Promotion and Predictability of Efficacy by In Vitro Physiological Traits. Int. J. Microbiol..

[23] Miao G.P., Han J., Wang C.R., Zhang K.G., Wang S. (2018). Chang Growth Inhibition and Induction of Systemic Resistance against *Pythium aphanidermatum* by *Bacillus simplex* Strain HS-2. Biocontrol. Sci. Technol..

[24] Ongena M., Jourdan E., Adam A., Paquot M., Brans A., Joris B., Arpigny J.L., Thonart P. (2007). Surfactin and Fengycin Lipopeptides of Bacillus Subtilis as Elicitors of Induced Systemic Resistance in Plants. Environ. Microbiol..

[25] Patel S., Gupta R.S. (2020). A Phylogenomic and Comparative Genomic Framework for Resolving the Polyphyly of the Genus Bacillus: Proposal for Six New Genera of Bacillus Species, Peribacillus Gen. Nov., Cytobacillus Gen. Nov., Mesobacillus Gen. Nov., Neobacillus Gen. Nov., Metabacillus Gen. Nov. and Alkalihalobacillus Gen. Nov. Int. J. Syst. Evol. Microbiol..

[26] Parte A.C., Carbasse J.S., Meier-Kolthoff J.P., Reimer L.C., Göker M. (2020). List of Prokaryotic Names with Standing in Nomenclature (LPSN) Moves to the DSMZ. Int. J. Syst. Evol. Microbiol..

[27] Seuylemezian A., Ott L., Wolf S., Fragante J., Yip O., Pukall R., Schumann P., Vaishampayan P. (2020). *Bacillus glennii* Sp. Nov. and *Bacillus saganii* Sp. Nov., Isolated from the Vehicle Assembly Building at Kennedy Space Center Where the Viking Spacecraft Were Assembled. Int. J. Syst. Evol. Microbiol..

[28] Wainwright M., Wickramasinghe N.C., Narlikar J.V., Rajaratnam P. (2003). Microorganisms Cultured from Stratospheric Air Samples Obtained at 41 Km. FEMS Microbiol. Lett..

[29] Ma K., Yin Q., Chen L., Lai Q., Xu Y. (2018). Bacillus Acanthi Sp. Nov., Isolated from the Rhizosphere Soil of a Mangrove Plant Acanthus Ilicifolius. Int. J. Syst. Evol. Microbiol..

[30] Liu Y., Yu M., Zhang X.H. (2018). *Bacillus alkalitolerans* Sp. Nov., Isolated from Marine Sediment near a Hydrothermal Vent. Int. J. Syst. Evol. Microbiol..

[31] Yumoto I., Hirota K., Yamaga S., Nodasaka Y., Kawasaki T., Matsuyama H., Nakajima K. (2004). *Bacillus asahii* Sp. Nov., a Novel Bacterium Isolated from Soil with the Ability to Deodorize the Bad Smell Generated from Short-Chain Fatty Acids. Int. J. Syst. Evol. Microbiol..

[32] Kuisiene N., Raugalas J., Spröer C., Kroppenstedt R.M., Chitavichius D. (2008). *Bacillus butanolivorans* Sp. Nov., a Species with Industrial Application for the Remediation of n-Butanol. Int. J. Syst. Evol. Microbiol..

[33] Rodríguez M., Reina J.C., Sampedro I., Llamas I., Martínez-Checa F. (2022). *Peribacillus castrilensis* Sp. Nov.: A Plant-Growth-Promoting and Biocontrol Species Isolated From a River Otter in Castril, Granada, Southern Spain. Front. Plant Sci..

[34] Feng L., Liu D., Sun X., Wang G., Li M. (2016). *Bacillus Cavernae* Sp. Nov. Isolated from Cave Soil. Int. J. Syst. Evol. Microbiol..

[35] Zhang L., Wu G.L., Wang Y., Dai J., Fang C.X. (2011). *Bacillus deserti* Sp. Nov., a Novel Bacterium Isolated from the Desert of Xinjiang, China. Antonie Leeuwenhoek Int. J. Gen. Mol. Microbiol..

[36] Zhang Y.Z., Chen W.F., Li M., Sui X.H., Liu H.C., Zhang X.X., Chen W.X. (2012). *Bacillus endoradicis* Sp. Nov., an Endophytic Bacterium Isolated from Soybean Root. Int. J. Syst. Evol. Microbiol..

[37] Jiang L., Jung W.Y., Li Z., Lee M.-K., Kang S.W., Lee J.-S., Jung H., Hur T.-Y., Kim H.B., Kim J.-K. (2019). *Peribacillus faecalis* Sp. Nov., a Moderately Halophilic Bacterium Isolated from the Faeces of a Cow. Int. J. Syst. Evol. Microbiol..

[38] Delaporte B., Sasson A. (1967). Étude de Bactéries Des Sols Arides Du Maroc: *Brevibacterium halotolerans* n. Sp. et *Brevibacterium Frigoritolerans* n. Sp.. Compte Rendu L’académie Sci..

[39] Montecillo J.A.V., Bae H. (2022). Reclassification of *Brevibacterium frigoritolerans* as *Peribacillus frigoritolerans* Comb. Nov. Based on Phylogenomics and Multiple Molecular Synapomorphies. Int. J. Syst. Evol. Microbiol..

[40] Kämpfer P., Busse H.J., McInroy J.A., Glaeser S.P. (2015). *Bacillus gossypii* Sp. Nov., Isolated from the Stem of Gossypium Hirsutum. Int. J. Syst. Evol. Microbiol..

[41] Li J., Yang G., Wu M., Zhao Y., Zhou S. (2014). *Bacillus huizhouensis* Sp. Nov., Isolated from a Paddy Field Soil. Antonie Leeuwenhoek Int. J. Gen. Mol. Microbiol..

[42] Lim J.M., Jeon C.O., Lee J.R., Park D.J., Kim C.J. (2007). *Bacillus kribbensis* Sp. Nov., Isolated from a Soil Sample in Jeju, Korea. Int. J. Syst. Evol. Microbiol..

[43] Liu B., Liu G.H., Zhu Y.J., Wang J.P., Che J.M., Chen Q.Q., Chen Z. (2016). *Bacillus loiseleuriae* Sp. Nov., Isolated from Rhizosphere Soil from a Loiseleuria Plant. Int. J. Syst. Evol. Microbiol..

[44] Afouda P., Dubourg G., Cadoret F., Fournier P.E., Raoult D. (2017). ‘*Bacillus massiliglaciei*’, a New Bacterial Species Isolated from Siberian Permafrost. New Microbes New Infect..

[45] Heyrman J., Logan N.A., Rodríguez-Díaz M., Scheldeman P., Lebbe L., Swings J., Heyndrickx M., De Vos P. (2005). Study of Mural Painting Isolates, Leading to the Transfer of “*Bacillus maroccanus*” and “*Bacillus carotarum*” to Bacillus Simplex, Emended Description of Bacillus Simplex, Re-Examination of the Strains Previously Attributed to “*Bacillus macroides*” and Description of *Bacillus muralis* Sp. Nov. Int. J. Syst. Evol. Microbiol..

[46] Priest F.G., Goodfellow M., Todd C. (1988). A Numerical Classification of the Genus Bacillus. J. Gen. Microbiol..

[47] Narsing Rao M.P., Dhulappa A., Banerjee A., Thamchaipenet A. (2022). Transfer of *Bacillus tepidiphilus* Narsing Rao et al. 2021 to the Genus *Peribacillus* as *Peribacillus tepidiphilus* Comb. Nov. Arch. Microbiol..

[48] Martínez-Hidalgo P., Flores-Félix J.D., Sánchez-Juanes F., Rivas R., Mateos P.F., Regina I.S., Peix Á., Martínez-Molina E., Igual J.M., Velázquez E. (2021). Identification of Canola Roots Endophytic Bacteria and Analysis of Their Potential as Biofertilizers for Canola Crops with Special Emphasis on Sporulating Bacteria. Agronomy.

[49] Chandra P., Khobra R., Sundha P., Sharma R.K., Jasrotia P., Chandra A., Singh D.P., Singh G.P. (2021). Plant Growth Promoting Bacillus-Based Bio Formulations Improve Wheat Rhizosphere Biological Activity, Nutrient Uptake and Growth of the Plant. Acta Physiol. Plant.

[50] Cochard B., Giroud B., Crovadore J., Chablais R., Arminjon L., Lefort F. (2022). Endophytic PGPR from Tomato Roots: Isolation, In Vitro Characterization and In vivo Evaluation of Treated Tomatoes (*Solanum lycopersicum* L.). Microorganisms.

[51] Hassen A.I., Labuschagne N. (2010). Root Colonization and Growth Enhancement in Wheat and Tomato by Rhizobacteria Isolated from the Rhizoplane of Grasses. World J. Microbiol. Biotechnol..

[52] Gutiérrez-Luna F.M., López-Bucio J., Altamirano-Hernández J., Valencia-Cantero E., De La Cruz H.R., Macías-Rodríguez L. (2010). Plant Growth-Promoting Rhizobacteria Modulate Root-System Architecture in Arabidopsis Thaliana through Volatile Organic Compound Emission. Symbiosis.

[53] Schwartz A.R., Ortiz I., Maymon M., Herbold C.W., Fujishige N.A., Vijanderan J.A., Villella W., Hanamoto K., Diener A., Sanders E.R. (2013). Bacillus Simplex—A Little Known Pgpb with Anti-Fungal Activity—Alters Pea Legume Root Architecture and Nodule Morphology When Coinoculated with Rhizobium Leguminosarum Bv. Viciae. Agronomy.

[54] Al-Sman K.M., Abo-Elyousr K., Eraky A., El-Zawahry A. (2019). Potential Activities of *Bacillus simplex* as a Biocontrol Agent against Root Rot of *Nigella sativa* Caused by *Fusarium camptoceras*. Egypt. J. Biol. Pest Control.

[55] Hansen V., Bonnichsen L., Nunes I., Sexlinger K., Lopez S.R., van der Bom F.J.T., Nybroe O., Nicolaisen M.H., Jensen L.S. (2020). Seed Inoculation with *Penicillium bilaiae* and *Bacillus simplex* Affects the Nutrient Status of Winter Wheat. Biol. Fertil. Soils.

[56] Erturk Y., Ercisli S., Haznedar A., Cakmakci R. (2010). Effects of Plant Growth Promoting Rhizobacteria (PGPR) on Rooting and Root Growth of Kiwifruit (*Actinidia deliciosa*) Stem Cuttings. Biol. Res..

[57] Senger M., Moresco E., Dalbosco M., Santin R., Inderbitzin P., Barrocas E.N. (2022). Methods to Quantify Bacillus Simplex-Based Inoculant and Its Effect as a Seed Treatment on Field-Grown Corn and Soybean in Brazil. J. Seed Sci..

[58] Rezakhani L., Motesharezadeh B., Tehrani M.M., Etesami H., Mirseyed Hosseini H. (2019). Phosphate–Solubilizing Bacteria and Silicon Synergistically Augment Phosphorus (P) Uptake by Wheat (*Triticum aestivum* L.) Plant Fertilized with Soluble or Insoluble P Source. Ecotoxicol. Environ. Saf..

[59] Sözer Bahadir P., Liaqat F., Eltem R. (2018). Plant Growth Promoting Properties of Phosphate Solubilizing Bacillus Species Isolated from the Aegean Region of Turkey. Turk. J. Botany.

[60] Yao Z., Chen Y., Luo S., Wang J., Zhang J., Zhang J., Tian C., Tian L. (2022). Culturable Screening of Plant Growth-Promoting and Biocontrol Bacteria in the Rhizosphere and Phyllosphere of Wild Rice. Microorganisms.

[61] Contreras-Liza S.E., Sanchez L.L., Davila D.E.Z. (2017). Agronomical Performance of Potato (*Solanum tuberosum* L.) Cv. “Unica” under Inoculation with Native Rhizobacteria and Application of Acetyl Salicylic Acid. Rev. Cienc. Agrovet..

[62] Mesanza N., Crawford B.D., Coulson T.J.D., Iturritxa E., Patten C.L. (2019). Colonization of *Pinus radiata* D. Don Seedling Roots by Biocontrol Bacteria *Erwinia billingiae* and *Bacillus simplex*. Forests.

[63] Parikh L., Eskelson M.J., Adesemoye A.O. (2018). Relationship of in Vitro and in Planta Screening: Improving the Selection Process for Biological Control Agents against Fusarium Root Rot in Row Crops. Arch. Phytopathol. Plant Prot..

[64] Gu Y.Q., Mo M.H., Zhou J.P., Zou C.S., Zhang K.Q. (2007). Evaluation and Identification of Potential Organic Nematicidal Volatiles from Soil Bacteria. Soil Biol. Biochem..

[65] Allioui N., Driss F., Dhouib H., Jlail L., Tounsi S., Frikha-Gargouri O. (2021). Two Novel Bacillus Strains (Subtilis and Simplex Species) with Promising Potential for the Biocontrol of Zymoseptoria Tritici, the Causal Agent of Septoria Tritici Blotch of Wheat. Biomed Res. Int..

[66] Adiyaman T., Schisler D.A., Slininger P.J., Sloan J.M., Jackson M.A., Rooney A.P. (2011). Selection of Biocontrol Agents of Pink Rot Based on Efficacy and Growth Kinetics Index Rankings. Plant Dis..

[67] des Essarts Y.R., Cigna J., Quêtu-Laurent A., Caron A., Munier E., Beury-Cirou A., Hélias V., Faure D. (2016). Biocontrol of the Potato Blackleg and Soft Rot Diseases Caused by Dickeya Dianthicola. Appl. Environ. Microbiol..

[68] Manetsberger J., Caballero Gómez N., Benomar N., Christie G., Abriouel H. (2023). Antimicrobial profile of the culturable olive sporobiota and its potential as a source of biocontrol agents for major phytopathogens in olive agriculture. Pest Manag. Sci..

[69] Mesanza N., Iturritxa E., Patten C.L. (2016). Native Rhizobacteria as Biocontrol Agents of Heterobasidion Annosum s.s. and Armillaria Mellea Infection of Pinus Radiata. Biol. Control.

[70] Iturritxa E., Trask T., Mesanza N., Raposo R., Elvira-Recuenco M., Patten C.L. (2017). Biocontrol of Fusarium Circinatum Infection of Young Pinus Radiata Trees. Forests.

[71] Xing Z., Wu X., Zhao J., Zhao X., Zhu X., Wang Y., Fan H., Chen L., Liu X., Duan Y. (2020). Isolation and Identification of Induced Systemic Resistance Determinants from Bacillus Simplex Sneb545 against *Heterodera glycines*. Sci. Rep..

[72] Kang W., Zhu X., Wang Y., Chen L., Duan Y. (2018). Transcriptomic and Metabolomic Analyses Reveal That Bacteria Promote Plant Defense during Infection of Soybean Cyst Nematode in Soybean. BMC Plant. Biol..

[73] Kang W.S., Chen L.J.L.J., Wang Y.Y., Zhu X.F., Liu X.Y., Fan H., Duan Y.X. (2020). Bacillus Simplex Treatment Promotes Soybean Defence against Soybean Cyst Nematodes: A Metabolomics Study Using GC-MS. PLoS ONE.

[74] Khayi S., des Essarts Y.R., Mondy S., Moumni M., Hélias V., Beury-Cirou A., Faure D. (2015). Draft Genome Sequences of the Three Pectobacterium-Antagonistic Bacteria Pseudomonas Brassicacearum PP1-210F and PA1G7 and Bacillus Simplex BA2H3. Genome. Announc..

[75] Valentine N., Bolton Jr H., Kingsley M., Drake G., BalkwilF D., Plymale A. (1996). Biosorption of Cadmium, Cobalt, Nickel, and Strontium by a *Bacillus simplex* Strain Isolated from the Vadose Zone. J. Ind. Microbiol. Biotechnol..

[76] Chamekh A., Kharbech O., Driss-Limam R., Fersi C., Khouatmeya M., Chouari R. (2021). Evidences for Antioxidant Response and Biosorption Potential of *Bacillus simplex* Strain 115 against Lead. World J. Microbiol. Biotechnol..

[77] Teng Z., Shao W., Zhang K., Huo Y., Li M. (2019). Characterization of Phosphate Solubilizing Bacteria Isolated from Heavy Metal Contaminated Soils and Their Potential for Lead Immobilization. J. Environ. Manag..

[78] Arce-Inga M., González-Pérez A.R., Hernandez-Diaz E., Chuquibala-Checan B., Chavez-Jalk A., Llanos-Gomez K.J., Leiva-Espinoza S.T., Oliva-Cruz S.M., Cumpa-Velasquez L.M. (2022). Bioremediation Potential of Native Bacillus Sp. Strains as a Sustainable Strategy for Cadmium Accumulation of Theobroma Cacao in Amazonas Region. Microorganisms.

[79] Seo J.S., Keum Y.S., Li Q.X. (2009). Bacterial Degradation of Aromatic Compounds. Int. J. Environ. Res. Public Health.

[80] Mandree P., Masika W., Naicker J., Moonsamy G., Ramchuran S., Lalloo R. (2021). Bioremediation of Polycyclic Aromatic Hydrocarbons from Industry Contaminated Soil Using *Indigenous bacillus* spp.. Processes.

[81] Yang Q., Yang T., Shi Y., Xin Y., Zhang L., Gu Z., Li Y., Ding Z., Shi G. (2021). The Nitrogen Removal Characterization of a Cold-Adapted Bacterium: *Bacillus simplex* H-b. Bioresour. Technol..

[82] Silva V., Mol H.G.J., Zomer P., Tienstra M., Ritsema C.J., Geissen V. (2019). Pesticide Residues in European Agricultural Soils—A Hidden Reality Unfolded. Sci. Total Environ..

[83] Erguven G.O., Yildirim N. (2016). Efficiency of Some Soil Bacteria for Chemical Oxygen Demand Reduction of Synthetic Chlorsulfuron Solutions under Agiated Culture Conditions. Cell Mol. Biol..

[84] Kansour M.K., Al-Mailem D.M. (2023). Bioremediation of Two Oil-Contaminated Kuwaiti Hyper-Saline Soils by Cross Bioaugmentation and the Role of Indigenous Halophilic/Halotolerant Hydrocarbonoclastic Bacteria. Environ. Technol. Innov..

[85] Mani P., Sivakumar P., Balan S.S. (2016). Economic Production and Oil Recovery Efficiency of a Lipopeptide Biosurfactant from a Novel Marine Bacterium *Bacillus simplex*. Achiev. Life Sci..

[86] European Parliament and the Council of the European Union Regulation (2009). (EC) No 1107/2009 of the European Parliament and the Council of 21 October 2009 Concerning the Placing of Plant Protection Products on the Market and Repealing Council Directives/117/EEC and 91/414/EEC. Off. J. Eur. Union.

[87] European Commission (2022). Commission Regulation (EU) 2022/1438 of 31 August 2022 Annex II to Regulation (EC) No 1107/2009 of the European Parliament and of the Council as Regards Specific Criteria for the Approval of Active Substances That Are Micro-Organisms. Off. J. Eur. Union.

[88] European Commission (2022). Commission Regulation (EU) 2022/1439 of 31 August 2022 Amending Regulation (EU) No 283/2013 as Regards the Information to Be Submitted for Active and the Specific Data Requirements for Micro-Organisms. Off. J. Eur. Union.

[89] European Commission (2013). Commission Regulation (EU) No 283/2013 of 1 March 2013 Setting out the Data Requirements for Active Substances, in Accordance with Regulation (EC) No 1107/2009 of the European Parliament and of the Council Concerning the Placing of Plant Protection Products on the Market. Off. J. Eur. Union.

[90] European Commission (2022). Commission Regulation (EU) 2022/1440 of 31 August 2022 Regulation (EU) No 284/2013 as Regards the Information to Be Submitted for Plant Protection Products and the Specific Data Requirements for Plant Protection Products Containing Micro-Organisms. Off. J. Eur. Union.

[91] European Commission (2013). Commission Regulation (EU) No 284/2013 of 1 March 2013 Setting out the Data Requirements for Plant Protection Products, in Accordance with Regulation (EC) of the European Parliament and of the Council Concerning the Placing of Plant Protection Products on the Market. Off. J. Eur. Union.

[92] European Commission (2011). Commission Regulation (EU) No 546/2011 of 10 June 2011 Regulation (EC) No 1107/2009 of the European Parliament and of the Council as regards Uniform Principles for Evaluation and Authorisation of Plant Protection Products. Off. J. Eur. Union.

[93] European Commission (2022). Commission Regulation (EU) 2022/1441 of 31 August 2022 Regulation (EU) No 546/2011 as Regards Specific Uniform Principles for Evaluation and Authorisation of Plant Protection Products Containing Micro-Organisms. Off. J. Eur. Union.

